# Determinants of modern contraceptive use among married women of reproductive age in ethiopia: a cross-sectional analysis of the 2019 Ethiopian mini demographic and health survey

**DOI:** 10.1038/s41598-025-19153-w

**Published:** 2025-10-09

**Authors:** Amanuel Mengistu Merera, Mesfin Esayas Lelisho, Wegayehu Enbeyle Sheferaw

**Affiliations:** 1 Department of Epidemiology and Biostatistics, College of Public Health, Jimma University, Jimma, Ethiopia; 2 Department of Statistics, College of Natural and Computational Sciences, Mizan-Tepi University, Tepi, Ethiopia; 3 Health Research Institute, University of Canberra, Australian Capital Territory (ACT), Canberra, Australia

**Keywords:** Modern contraceptive, Married women, Reproductive age, Ethiopia, Mini-DHS, Health services, Public health

## Abstract

In Ethiopia, despite efforts to increase modern contraceptive use, prevalence remains relatively low. This study aimed to identify factors associated with modern contraceptive use among married women of reproductive age in Ethiopia. We performed a cross-sectional study analyzing 5,684 weighted samples using SPSS version 20. We employed logistic regression model to examine the association between sociodemographic factors and modern contraceptive use. The adjusted odds ratios and 95% confidence intervals (CI) were calculated. Among currently married women, 38.2% were using modern contraceptives. Injectable contraceptives were the most popular method (60.1%), followed by implants (23%), pills (6.7%), IUDs (5.4%), LAM (2.8%), and others (2.0%). Logistic regression analysis revealed that, women age 20–24 (AOR = 4.044, 95% CI: 2.883–5.674), urban residence (AOR = 1.32, 95% CI: 1.09–1.59), no education (AOR = 0.548, 95% CI: 0.419–0.718), no knowledge of modern contraceptive (AOR = 0.039, 95% CI: 0.014–0.107), and poor wealth index (AOR = 0.45, 95% CI: 0.38–0.53) were significant associated with modern contraceptive use. To enhance family planning in Ethiopia, interventions should increase access to diverse contraceptive methods, especially in rural areas. Community awareness initiatives and government programs can help individuals make informed reproductive health choices. Public education through media, community-based efforts, and effective counseling is also essential.

## Introduction

Unintended pregnancy remains a significant global health challenge, particularly in developing countries like Ethiopia^1,2^. It contributes to maternal morbidity and mortality, limits educational and economic opportunities for women, and fuels population growth^2–4^. Family planning, particularly through modern contraceptive use, is a crucial strategy for addressing this issue and improving reproductive health worldwide^5^.

While global contraceptive use has increased, particularly in regions like Asia and Latin America, progress in Sub-Saharan Africa has been slower. Between 1990 and 2015, global modern contraceptive use rose from 54 to 57.4%, while in Africa, it increased from 23.6 to 28.5%^4,6,7^. Ethiopia, a populous African nation, has a high fertility rate of 4.2 children per woman, among the highest globally^8^. Despite efforts to increase contraceptive use in Ethiopia, various studies report that its prevalence remains relatively low^8–17^.

Previous studies have identified age, residence, wealth, education, parity, early marriage, and fertility preference as key factors influencing contraceptive use in 61 countries^4,9,13,14,16,18–21^. Additionally, a Tanzanian study highlighted women’s empowerment, age disparities with partners, and desired family size as significant predictors^22^. Additional factors influencing contraceptive use include cultural norms, access to healthcare, and male partner involvement^23–25^. Cultural beliefs can shape attitudes towards family planning, while accessible healthcare services and supportive male partners can positively impact women’s contraceptive choices^16,26–29^.

To address this challenge, Ethiopia’s health sector transformation plan aims to increase contraceptive prevalence to 55% and reduce unmet needs to 10% by 2020^30,31^. However, realizing this vision necessitates a comprehensive understanding of the factors influencing contraceptive use. By identifying the underlying sociodemographic, cultural, and behavioral factors associated with contraceptive uptake, policymakers and program implementers can tailor interventions to effectively address the specific needs of the population.

This study, utilizing data from the 2019 Ethiopian Demographic and Health Survey (EDHS), aims to contribute to this understanding by investigating the prevalence and determinants of modern contraceptive use among married women in Ethiopia. By examining the sociodemographic and behavioral factors associated with contraceptive use, this research seeks to inform evidence-based interventions that can improve family planning uptake and ultimately lead to better reproductive health outcomes in the country.

## Data and methods

### Study design and setting

A community-based cross-sectional study was conducted in Ethiopia from March 21 to June 28, 2019. As the second-most populous country in Africa, Ethiopia has a population of approximately 105,350,020 people^32^ and ranks as the tenth-largest country in terms of area, covering 1,100,000 km². The country is organized into eleven geographical or administrative divisions, including nine regional states and two city administrations, with Addis Ababa serving as the capital. This study utilized data from the 2019 Ethiopian Mini Demographic and Health Survey (EMDHS), which is the second instance of such a survey conducted in Ethiopia. The dataset focused on married women aged 15 to 49 years. The survey was implemented under the auspices of the Ministry of Health, coordinated by the Ethiopian Public Health Institute (EPHI).

During the data collection, interviewers used tablet computers to record responses. These tablets featured Bluetooth technology, enabling remote transfer of electronic files, such as assignment sheets and completed questionnaires, via computer-assisted personal interviewing (CAPI). The electronic data collection system for the 2019 EMDHS was developed using a mobile version of the Census and Survey Processing program by the DHS Program^33^.

## Population and sampling procedures

The sample for the 2019 Ethiopian Mini Demographic and Health Survey (EMDHS) was selected using a two-stage cluster design. In the first phase, census enumeration areas (EAs) served as sampling units, stratified by region and urban-rural status. The sample included 305 EAs, comprising 93 urban and 212 rural areas.

**Fig. 1:**
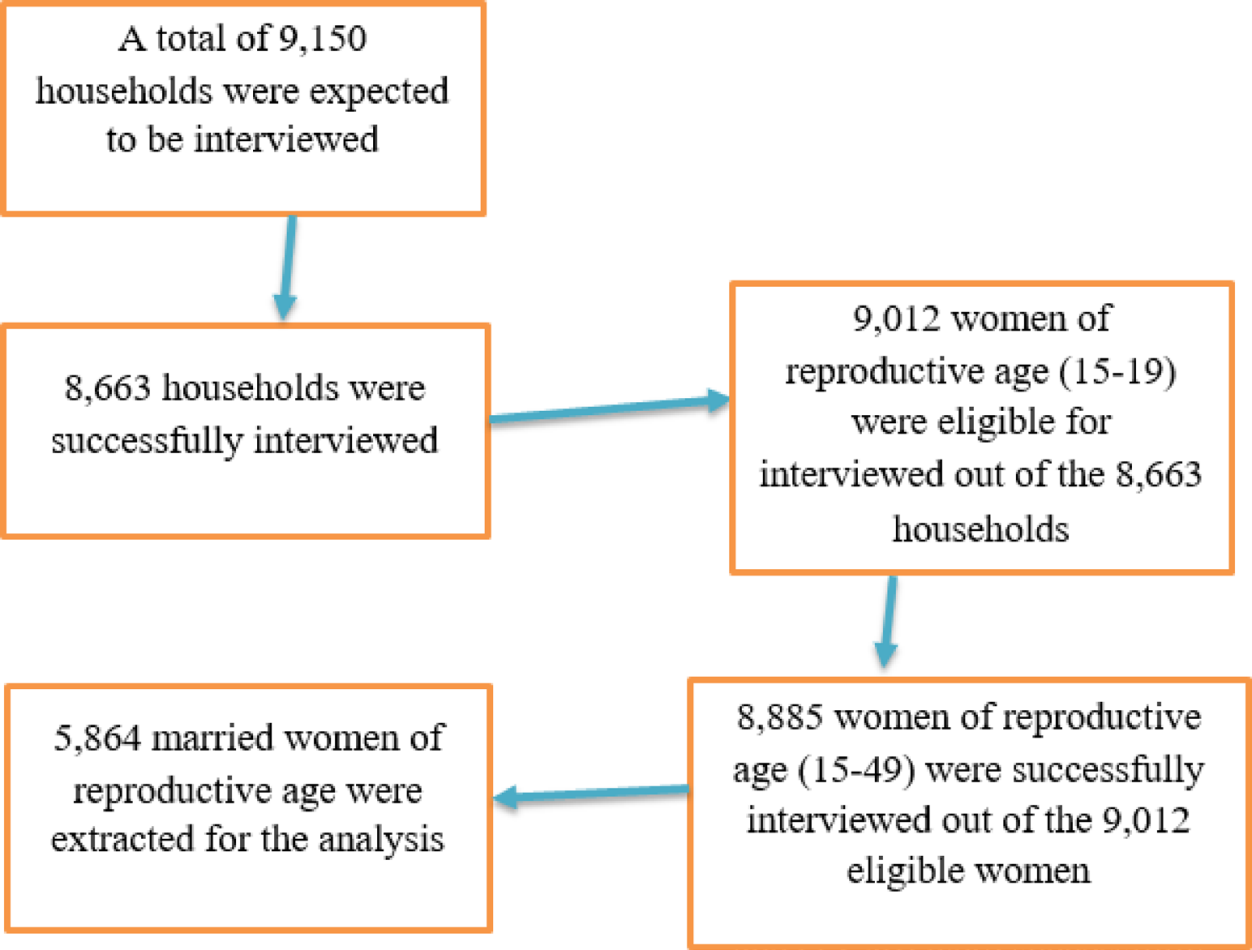
Schematic presentation of how the study sample was selected.

In the second phase, households were sampled using equal probability systematic sampling proportional to the size of each EA. From March 21 to June 28, 2019, a complete household listing was conducted in the selected EAs. A total of 9,150 households were identified, of which 8,794 were occupied, and interviews were successfully completed in 8,663 households. Within these households, 9,012 eligible women aged 15 to 49 were identified, and interviews were conducted with 8,885 women^34^. The analysis for this study focused on married women, resulting in a final dataset of 5,864 eligible married women from the 2019 EMDHS (Fig. 1).

## Exclusion criteria

Participants who were pregnant or sexually inactive at the time of the survey were excluded to ensure accurate assessment of modern contraceptive use. Additionally, observations with missing data on key study variables were removed to maintain data quality and validity.

## Data quality control

Data collectors were given training in order to ensure that, the data was of high quality. Data quality control and fieldwork coordination were also taught to regional coordinators, field supervisors, and CAPI (computer-assisted personal interview) supervisors. The data collection was overseen by the research investigators on a daily basis. A protocol was written and given to data collectors that governs the survey’s design, execution, and administration. Interviews were conducted in quiet, pleasant places at convenient times after data collectors were briefed. Furthermore, participants were encouraged to provide honest responses by describing the study’s purpose and significance, as well as ensuring the confidentiality of the data they would provide. Completed surveys were checked for completeness and consistency on a daily basis.

## Variables in the study

### Outcome variable

The primary outcome of this study was modern contraceptive use among married women in Ethiopia. In this study, the responses can be 1 = modern contraceptive users i.e. women currently using a modern contraceptive method; 0 = non-modern contraceptive users i.e. women using traditional or folkloric methods, or not using any method. Previous research^34–36^ have detailed the sorts of contraceptives classified as modern, traditional, or folklore.

## Independent variables

The study considered the following independent variables, selected based on previous research^4,11,16,19,24,27,34–36^ and their potential influence on modern contraceptive use among married women were: women’s age (15–19,20–24,25–29,30–34,35–39,40–44, and 45–489), religion (Orthodox, Catholic, Protestant, Muslim, and Others), women’s educational level (No education, Primary, Secondary, and Higher), and place of residence were all found to be associated with modern contraceptive practice among married women, according to the literature review. The DHS classified households’ wealth into four categories: poorest, poorer, middle, rich, and richer. We were then recoded as poor (poorest and poorer), middle, and rich (richer and richest). Number of living children (no child, one to two children, three to four children, and five or more children), province (Tigray, Afar, Amhara, Oromia, Somalia, Benishangul Gumuz, SNNPR, Gambela, Harari), and awareness of modern contraceptive methods (had knowledge about modern contraceptive methods and hand no knowledge about modern contraceptives).

## Knowledge and awareness

- Awareness of modern contraceptive methods (Yes, No).

### Data processing and analysis

In the analysis of DHS data, sampling weights were utilized for each individual interview unit to account for variations in selection probability and interview occurrences among cases in the sample, which may arise from the study design, random factors, or adjustments for differing response rates. Applying sample weights is essential to ensure an accurate representation of the survey data. The data were also verified for completeness, cleaned to remove any missing values, and recoded as necessary.

Data collected for this study were entered and analyzed using SPSS Version 20. The analysis involved descriptive statistics and logistic regression models. Descriptive statistics, including percentages, were utilized to explain the study variables. To explore the independent relationships between demographic, socio-economic factors, and the use of modern contraception among married women, a chi-square test was performed, with a significance level set at *p* < 0.05.

Bivariate logistic regression was conducted to identify candidate variables for the multivariable analysis, using a p-value threshold of less than 0.2. Prior to developing the final model, the variance inflation factor (VIF) was calculated to assess multicollinearity among the selected variables. The study employed a logistic regression model to account for the complexities of the sampling design and the heterogeneity of observations within the same cluster, in line with the data collection strategy used for the Demographic and Health Surveys (DHS).

Results were presented as adjusted odds ratios with 95% confidence intervals. The model’s fit was assessed using Hosmer and Lemeshow’s test, with a p-value below 0.05 considered statistically significant for the final model.

### Operational definition of terms

- **Use**: Refers to the adoption of any modern contraceptive method aimed at spacing children and preventing unplanned pregnancies among individuals aged 15 to 49 years.
- **Current Modern Contraceptive Use**: This term describes individuals who reported using a modern contraceptive method at the time of the interview. This includes those who stated that they or their partner were currently utilizing one of the following methods: injection, intrauterine device (IUD), implant, lactational amenorrhea method (LAM), condom, female sterilization, or emergency contraception.
- **Knowledge of Contraceptives**: If a woman can use at least one type of modern contraceptive method, she can be considered knowledgeable about contraceptives.

## Results

### Study population characteristics

**Fig. 2:**
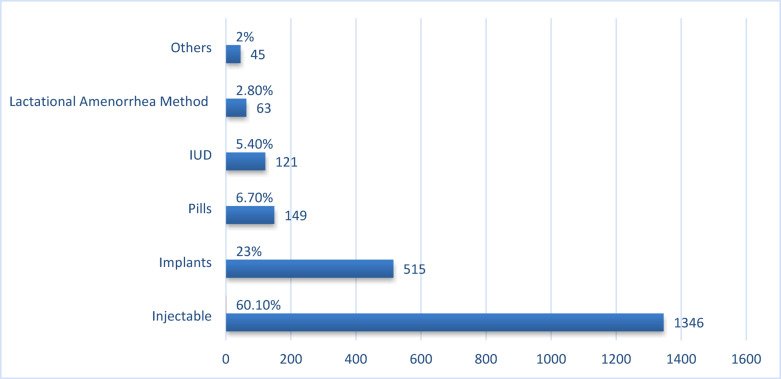
Married women currently using a modern contraceptive Methods.

This study aimed to explore the factors influencing modern contraceptive use among married women. The overall prevalence of contemporary contraceptive use was found to be 38.2% among the 5,684 married women surveyed, which is slightly lower than the national estimated prevalence rate. As shown in Tables 1, 2 and 239 (38.2%) of the participants reported using some form of contraception at the time of data collection. In Ethiopia, the most commonly used modern contraceptive methods among currently married women of reproductive age include injectables (60.1%), implants (23%), the lactational amenorrhea method (2.8%), and other methods (2.0%) as illustrated in (Fig. 2).

**Fig. 3:**
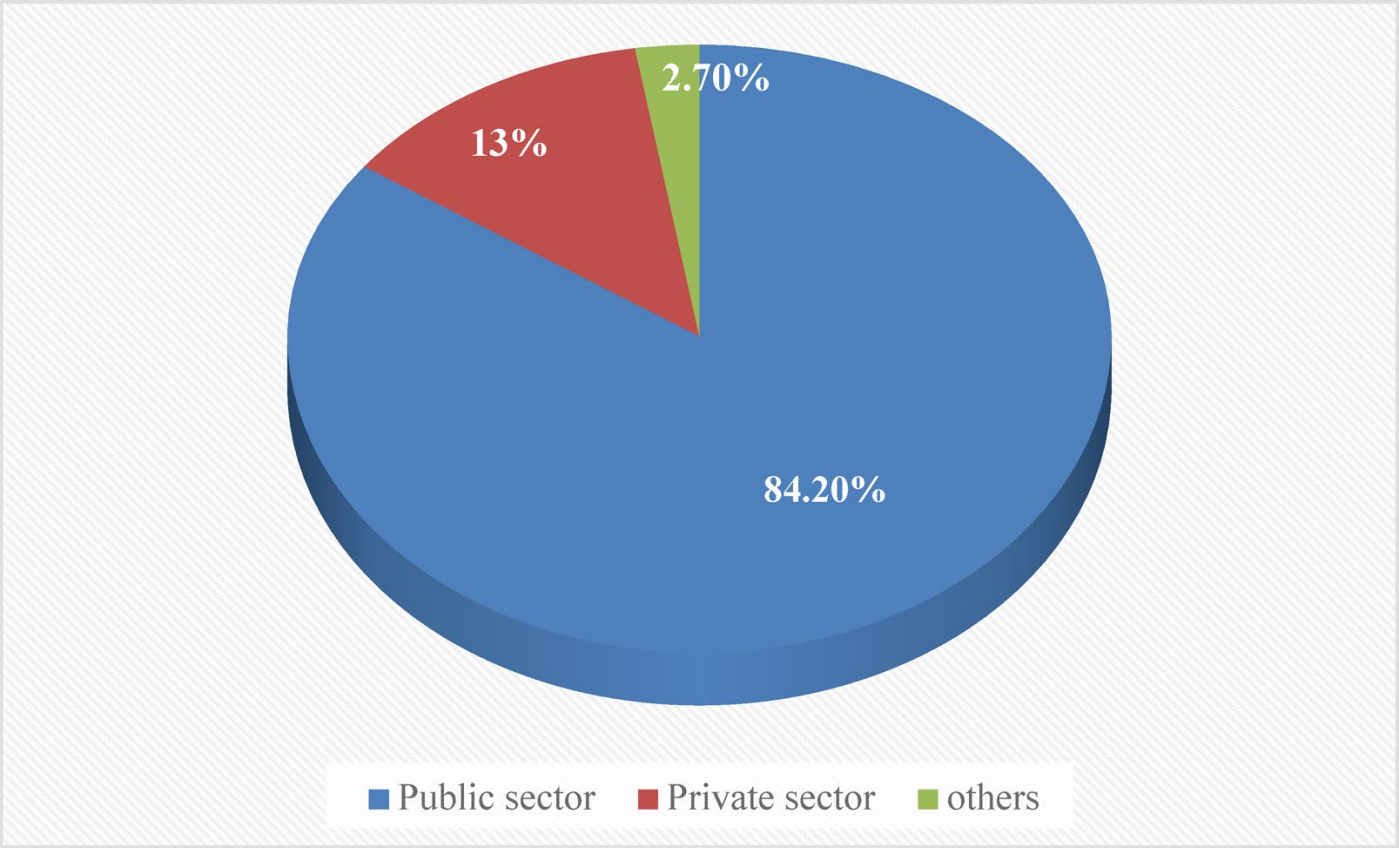
Source of modern contraceptive among women of reproductive age in Ethiopia.

Every married woman within the reproductive age group reported using at least one modern contraceptive method. The public sector served as the primary source of contemporary contraception, accounting for 84.2% of usage, followed by the private sector at 13% and other sources at 2.7%. Within the public sector, government health stations and health posts were the most frequently utilized sources (Fig. 3).

Results on the distribution of modern contraceptive use across the explanatory variables indicated in Table 1 shows that, modern contraceptive practice among married women according to women’s age categories 15–19, 20–24, 25–29, 30–34, 35–39, 40–44, 45–49 were 31.7%, 45.6%, 43.9%,41.2%, 36.6%, 28.2% and 18.9% respectively. Most of the women who used modern contraceptives were aged 20–24 (45.6%), but minimum numbers of modern contraceptive practice were observed in the age group of 45–49. This is may be due to the fact that the women fertility period is stopped at this age.

The number of married women who utilize modern contraception has likewise varied significantly across the region. As a result, the largest percentages of modern contraceptive use are seen in Amhara (51.4%), Addis Ababa (51%), SNNPR (40.7%), and Tigray (40.7%). (35.6%). Somalia, on the other hand, has the lowest rates of contraception use (3.3%). The percentage of people who use a contraception is practically identical in the left regions.

Prevalence of modern contraceptive user in terms of place of residence in rural and urban were 34.4% and 48.1% respectively. The modern contraceptive practice among married women of reproductive age according to their educational level reveals that 26.9% of no education, 48.0% of a Primary level, 50.3% of a Secondary level, and 57% of higher-level women were using some form of modern contraceptive.

Percentages of modern contraceptive among women of reproductive age in terms of family wealth index among Poor, middle and higher were 23%, 45.7%, and 50% respectively. The proportion of modern contractive practice among married women who had 1–2 children were 51.6% more experiencing modern contraceptive than a woman who had five or more children (25.5%). Furthermore, the proportion of modern contraceptive practice among women in terms of religion categories: Orthodox, Catholic, Protestant, Muslim and other were 50.6%, 50%, 46.9%, 24.1% and32.3% respectively. On the other hand, the percentage of modern contraceptive user among married women of reproductive age was highly observed (40.8%) with women who have knowledge about modern contraceptive than those who do not have knowledge about modern contraceptive (1%).

The analysis of modern contraceptive use across various explanatory variables, as shown in Table 1, reveals distinct patterns based on women’s age categories. The prevalence of modern contraceptive use among married women aged 15–19, 20–24, 25–29, 30–34, 35–39, 40–44, and 45–49 years was 31.7%, 45.6%, 43.9%, 41.2%, 36.6%, 28.2%, and 18.9%, respectively. The highest usage was observed among women aged 20–24 (45.64%), while the lowest was found in the 45–49 age group, likely due to the completion of the reproductive period at this age.

The utilization of modern contraception varied significantly by region. The highest rates of modern contraceptive use were recorded in Amhara (51.4%), Addis Ababa (51%), SNNPR (48.9%), and Tigray (39.5%), with Somalia showing the lowest at just 3.3%. The percentage of modern contraceptive users was similar across the neighboring regions.

Regarding place of residence, the prevalence of modern contraceptive use was 34.4% in rural areas compared to 48.1% in urban settings. The analysis also indicated that modern contraceptive usage varied by educational attainment, with 26.9% of women with no education, 48.0% with a primary education, 50.3% with secondary education, and 57.0% with higher education reporting the use of modern methods.

When considering family wealth index, the percentages of modern contraceptive use were 23% among poor women, 45.7% among those from middle-income households, and 50% among the wealthier group. Additionally, women with 1–2 children were more likely to utilize modern contraceptives (51.6%) compared to those with five or more children (25.5%).

The analysis of modern contraceptive practice by religious affiliation indicated that usage rates were 50.6% among Orthodox Christians, 50% among Catholics, 46.9% among Protestants, 24.1% among Muslims, and 32.3% among others. Furthermore, a notable 40.8% of women with knowledge about modern contraceptive methods reported using them, in stark contrast to just 1% of those without such knowledge.

**Table 1:** Distribution of demographic and Socio-economic factors affecting modern contraceptive use among married women of reproductive age in Ethiopia (March 21 to June 28, 2019) (*N* = 5,864).

Variables names	Categories	Modern contraceptive user among married women		Total	P-value
		Not user	User		
		Count (%)	Count (%)		
Women age	15–19	366(68.3)	170(31.7)	536	$\:\le\:$0.001
	20–24	555(54.4)	466(45.6)	1021	
	25–29	807(56.1)	631(43.9)	1438	
	30–34	592(58.8)	414(41.2)	1006	
	35–39	562(63.4)	325(36.6)	887	
	40–44	408(71.8)	160(28.2)	568	
	45–49	335(82.1)	73(17.9)	408	
Region	Tigray	270(60.5)	176(39.5)	446	$\:\le\:$0.001
	Afar	412(85.5)	70(14.5)	482	
	Amhara	337(48.6)	356(51.4)	693	
	Oromia	394(53.2)	346(46.8)	740	
	Somali	441(96.7)	15(3.3)	456	
	Benishangul	31457.6)	231(42.4)	545	
	SNNPR	362(51.1)	346(48.9)	708	
	Gambela	295(62.6)	176(37.4)	471	
	Harari	294(64.1)	165(35.9)	459	
	Addis Ababa	194(49.0)	202(51.0)	396	
	Dire Dawa	312(66.7)	156(33.3)	468	
Place of Resident	Urban	835(51.9)	773(48.1)	1608	$\:\le\:$0.001
	Rural	2790(65.6)	1466(34.4)	4256	
Women Education level	No education	2160(73.1)	794(26.9)	2954	$\:\le\:$0.001
	Primary	1011(52.0)	933(48.0)	1944	
	Secondary	289(49.7)	293(50.3)	582	
	Higher	165(43.0)	219(57.0)	384	
Wealth Index	Poor	1876(76.7)	569(23.3)	2445	$\:\le\:$0.001
	Middle	500(54.3)	420(45.7)	920	
	Rich	1249(50.0)	1250(50.0)	2499	
Number of living child	No child	575(70.8)	237(29.2)	812	$\:\le\:$0.001
	1–2 child	936(48.4)	998(51.6)	1934	
	3–4 child	889(60.4)	584(39.6)	1473	
	Five or more	1225(74.5)	420(25.5)	1645	
Religion	Orthodox	1030(49.4)	1055(50.6)	2085	$\:\le\:$0.001
	Catholic	20(50.0)	20(50.0)	40	
	Protestant	602(53.1)	532(46.9)	1134	
	Muslim	1931(75.9)	612(24.1)	2543	
	Other	42(67.7)	20(32.3)	62	
Knowledge of using contraceptive	No	380(99.0)	4(1.0)	384	$\:\le\:$0.001
	Yes	3245(59.2)	2235(40.8)	5480	

### Bivariate analysis

We used bivariate analysis to determine candidate variables for the multivariable binary logistic regression model before moving on to the multivariable binary logistic regression model. By assuming all significant factors in the bivariate analysis at a 20% significance level, multivariable binary logistic regressions were done. Fortunately, all of the factors in this study were statistically significant, thus the multivariate binary logistic regression model took them all into account again.

### Checking multi-collinearity

The level of multi-collinearity in a collection of available variables in the model is measured using a variable variance inflation factor (VIF). A high VIF shows whether or not the values used to detect the related explanatory variable are significantly collinear with other variables. VIF values larger than 10 are typically assumed to indicate multi-collinearity, while values greater than 2.5 in weaker models, such as logistic regression, may be cause for concern^37^. Based on the findings, all sets of variables in the current study have VIF values of less than 2.5, indicating that multi-collinearity is not an issue.

### Factors associated with modern contraceptive utilization among married women of reproductive age

In binary logistic regression seven variables had significant relationship with modern contraceptive user among married women of reproductive age. These factors include age of women, region, place of resident, women education level, wealth index of the household, number of living children, and knowledge of contraceptive use. However, there is no significant link between the use of modern contraception among married women of reproductive age and religion in multivariable logistic regression.

Women aged 15 to 19 years were 3.346 times more likely to use modern contraception than women aged 45 to 49 years (AOR = 3.327, 95% CI: 2.258–4.960) when other factors were held constant. Women who belong in the age group of 20–24, 25–29,30–34,35–39 and 40–44 years were 4.044, 3.672, 3.807, 2.976 and 1.982 times more likely to use modern contraceptive as compared to those women 45–49 years (AOR = 4.044, 95% CI: 2.883–5.674),25 to 29 (AOR = 3.672 95% CI: 5.689–5.014), 30 to 34 (AOR = 3.807 95% CI: 2.795–5.187), 35 to 39 (AOR = 2.976 95% CI: 2.183–4.057) and 40 to 44 (AOR = 1.892 95% CI: 1.420–2.767) respectively (See Table 2).

Married Women who resided in the Amhara region are 2.617 times more likely to practice modern contraceptive when compared with those residing in Dire Dawa controlling for other variables in the model (AOR = 2.617, 95% CI: 1.926–3.557). Married women of reproductive age who lived in Oromia region were 2.486 times more likely to practice modern contraceptive compared to women in Dire Dawa controlling for other variables in the model (AOR = 2.486, 95% CI: 1.864–3.317). married Women of reproductive age who lived in Benishangul Gumuz and SNNPR region were 2.171 and 2.457 times more likely to practice modern contraceptive compared to women in Dire Dawa controlling for other variables in the model (AOR = 2.171, 95% CI: 1.600-2.945 and AOR = 2.457, 95% CI: 1.797–3.358) respectively. Conversely, women who lived in Somali were 76.3% less likely to practice family planning compared to women in Dire Dawa (AOR = 0.237, 95% CI: 0.133–0.423).

Married Women who using modern contraceptive among reproductive age who resided in the urban areas were 32.1% more likely to practice modern contraceptive compared with those from the rural areas (AOR = 1.321, 95% CI:1.094–1.595). Modern contraceptive among Married Women of reproductive age who had no education were 45.2% (AOR = 0.548, 95% CI: 0.419–0.718) less likely to using modern contraceptive compared to women who had higher education.

Married Women who live in poor households were about 55.1% less likely to practice modern contraceptive than that of women who live in rich households (AOR = 0.449, 95% CI: 0.379–0.532) and married women who had medium wealth were about 20.1% less likely to practice modern contraceptive compared to women who were rich (AOR = 0.799, 95% CI: 0.661–0.966).

From Table 2, we also observed that married Women with no children had a significantly lower odds (or probability) of using modern contraception than women having five or more children. The odds of using contraceptives for a married woman with one or two children was 44.3% times more likely to use modern contraceptive among women of reproductive age compared to those who have five or more children (AOR = 1.443, 95% CI: 1.173–1.775) and married woman with three or four children was 25% times more likely to use modern contraceptive among women of reproductive age compared to those who have five or more children (AOR = 1.25, 95% CI: 1.044–1.499). Similarly, those married women of reproductive age who had no knowledge about modern contraceptive use were 61% less likely to use modern contraceptive compared to women who had knowledge about modern contraceptive use (AOR = 0.39, 95% CI: 0.014–0.107).

**Table 2:** Bivariate and multivariate analysis of factors associated with use of modern contraceptive among women of reproductive age in ethiopia, 2019 (*N* = 5864).

Variables	Categories	Modern contraceptive user		COR (95% CI)	AOR (95% CI)
		No	Yes		
Women Age	45–49 (ref)	335	73	1.0	1.0
	15–19	366	170	2.132(1.561–2.911)	3.346(2.258–4.960)*
	20–24	555	466	3.853(2.908–5.106)	4.044(2.883–5.674)*
	25–29	807	631	3.588(2.729–4.718)	3.672(2.689–5.014)*
	30–34	592	414	3.209(2.419–4.257)	3.807(2.795–5.187)*
	35–39	562	325	2.654(1.990–3.538)	2.976(2.183–4.057)*
	40–44	408	160	1.8(1.317–2.459)	1.982(1.420–2.767)*
Region	Dire Dawa(ref)	312	156	1.0	1.0
	Tigray	270	176	1.304(0.995–1.708)	1.092(0.784–1.522)
	Afar	412	70	0.340(0.247–0.467)	0.759(0.532–1.082)
	Amhara	337	356	2.113(1.657–2.694)	2.617(1.926–3.557)*
	Oromia	394	346	1.756(1.381–2.234)	2.486(1.864–3.317)*
	Somali	441	15	0.068(0.039–0.118)	0.237(0.133–0.423)*
	Benishangul	314	231	1.471(1.138–1.902)	2.171(1.600-2.945)*
	SNNPR	362	346	1.912(1.50-2.435)	2.457(1.797–3.358)*
	Gambela	295	176	1.193(0.913–1.560)	1.163(0.841–1.608)
	Harari	294	165	1.122(0.856–1.471)	0.916(0.681–1.232)
	Addis Ababa	194	202	2.082(1.581–2.742)	1.008(0.743–1.368)
Place of resident	Rural (ref)	2790	1466	1.0	1.0
	Urban	835	773	1.762(1.568–1.979)	1.321(1.094–1.595)*
Women educational level	Higher (ref)	165	219	1.0	1.0
	No education	2160	794	0.277(0.223–0.344)	0.548(0.419–0.718)*
	Primary	1011	933	0.695(0.558–0.867)	0.977(0.757–1.260)
	Secondary	289	219	0.764(0.589–0.990)	0.834(0.630–1.104)
Wealth index	Rich (ref)	1876	569	1.0	1.0
	Poor	500	420	0.303(0.268–0.342)	0.449(0.379–0.532)*
	Middle	1249	1250	0.839(0.721–0.977)	0.799(0.661–0.966)*
Number of child	Five or more (ref)	1225	420	1.0	1.0
	No child	575	237	1.202(0.997–1.450)	0.461(0.348–0.612)
	1–2 child	936	998	3.110(2.698–3.585)	1.443(1.173–1.775)*
	3–4 child	889	584	1.916(1.645–2.231)	1.251(1.044–1.499)*
Religion	Others (ref)	42	20	1.0	1.0
	Orthodox	1030	1055	2.151(1.254–3.689)	1.404(0.765–2.576)
	Catholic	20	20	2.1(0.928–4.754)	1.525(0.624–3.727)
	Protestant	602	532	1.856(1.076–3.201)	1.023(0.560–1.868)
	Muslim	1931	612	0.666(0.388–1.142)	0.693(0.378–1.270)
Knowledge of contraceptive use	Yes (ref)	3245	2235	1.0	1.0
	No	380	4	0.015(0.006–0.041)	0.039(0.014–0.107)*

* Significant at$\:p-value<0.05$, Hosmer and Lemeshow goodness of fit (p-value = 0.100).

## Discussion

In Ethiopia, this study looked at the use of modern contraception and the factors that influence it among married women of reproductive age (15–49 years). Women Modern contraceptive use was linked to age, region, women’s educational level, number of living children, place of residence, knowledge of modern contraceptive use, and household wealth index.

According to the findings of this study, 38.2%t of the participants were currently utilizing a modern contraceptive method, which is comparable to 37.8%^38^ and 37.9%^39^. It is slightly lower than the country’s expected prevalence level, but slightly higher than the prevalence for the rural community, which was recorded by the 2016 EDHS at 35%^40^, 31.7% in northwest Ethiopia^41^, 23.92% Misha district, South Ethiopia (It’s possible that the discrepancy is due to the study’s focus on married women solely)^42^, 21% in Ghana (It’s possible that the disparity is attributable to the study area’s small size and density)^43^. The variation is due to; the majority of the previous studies might be explained by the involvement of health extension workers in the awareness generation activity.

In this study, the age of women and the use of modern contraceptive methods were strongly associated. As women grew older from 15 to 34 years of age, the possibility of using modern contraceptive methods increased. This result was similar to the result of the study carried out in Nigeria, southern Ethiopia, Mali, Congo and Bangladesh^43–48^ respectively. The reason for this could be that in rural regions, this is the age group when most women want to engage in various activities to support their family needs, and hence wish to arrange their birth. As a result, they favor contraceptive methods. Another cause could be the greater sharing of experiences among friends, neighbors, and those age groups that now have women’s forums to talk about the subject.

This study found that women’s educational levels have an impact on their acceptance of modern contraceptives, with higher-educated women using them more frequently, which is corroborated by a prior study^49^. Women’s education levels climb, their wealth and prestige rise, and their desire to limit their offspring by utilizing modern contraception rises^50^. Obviously, education increases one’s capacity to earn riches and reputation; but, this competes with the option to have children, because in today’s economy, children are more resource consumers than creators for many years^50^. Higher-educated women have more access to health information, more independence in making their own informed decisions, and more ability to use health services, according to this theory. Women’s reproductive health benefits from education because it allows them to better understand and use the many methods of contraception that best meet their health needs.

Increased household wealth was found to be a strong predictor of increased usage of modern contraception. It was consistent with two previous studies conducted in Ethiopia, including a study conducted among rural women; it found that women who were in the top quintile were more likely to use modern contraception^51,52^. The usage of modern contraceptives can be influenced by wealth, either directly or indirectly. Women may be aware of the significance of birth control. In other circumstances, however, knowledge alone will not be sufficient. They should be able to afford transportation and customer service. The tradeoff connected with the time they spend commuting to and from healthcare institutions is also significant. This time can be used for domestic duties, farming, or other business-related activities.

The current study also revealed that place of residence is a significant factor contributing to modern contraceptive use. Women who lived in urban areas were more likely than those who lived in rural regions to use modern contraception (AOR = 1.321). The reality is that urban dwellers may have better access to family planning services, are more educated, and are more likely to utilize contraception than rural dwellers^4,53^. Similarly, residing in urban areas was found to increase the likelihood of utilizing modern contraceptive techniques compared to living in a rural region, which is consistent with earlier research^54,55^. This could be because urban dwellers are less inclined than their rural counterparts to travel large distances for medical care. In addition, we found regional disparities in the use of modern contraceptives, which is consistent with earlier findings^54^.

Theoretically, women with a large number of children are more likely than women without children to use modern contraceptive techniques. Contrary to popular belief, the higher the parity, the less contraception is used in this study. The study conducted in southern Ethiopia, similar outcomes have been found from^56^, Oromia Region Ethiopia^57^ and Ghana^20^ and the study carried out among 73 low and middle income countries^58^. The possible explanation could be that the women with a large number of children had no birth, as they were using modern contraceptive methods, and it could be more likely to be those who were not married, or the result of the study showed that the higher the family size, the higher likely is the unintended pregnancy, this is in line with the study carried out in Damote Gale, Southern Ethiopia and Debre-Markos Town, northwestern Ethiopia^59,60^. It’s possible that this is because families with a large number of children have already reached their ideal number of children, and any additional pregnancy is therefore likely to be unwanted.

According to a study conducted in Arba-Minch, women who are less knowledgeable about modern contraceptive methods are more likely to have unwanted pregnancies^61^. This could be explained by the fact that women who were unfamiliar with modern contraception methods were less likely to be aware of available, accessible, and safe contraception and other reproductive health services. As a result, the method is less likely to be used appropriately, which increases the chances of an unwanted pregnancy. In order to prevent undesired births, it is critical to raise the understanding of women of reproductive age about modern contraceptive.

According to the EMDHS, awareness of contraception is ubiquitous in Ethiopia, with 99% of the population knowing at least one method, yet regional disparities still exist^40^. In this study, 99.8% of participants have heard of modern contraceptives. In other studies, 97.2% were knowledgeable in the study on southern Ethiopia^42^, 98% in Ghana^43^ and 97.9% in Tanzania^62^. These findings point to a knowledge gap about the availability of modern contraceptive methods.

### Strengths and limitation of the study

#### Strengths and weakness

This research made use of extensive datasets and incorporated sample weighting in its analysis, which enhances the study’s statistical power and allows for broader applicability of the findings regarding modern contraceptive use among women of reproductive age. However, since the 2019 EMDHS is a cross-sectional study, it cannot establish causal relationships between various factors and the use of modern contraception. Additionally, the data relies on self-reported information from women, which may be influenced by recall bias. The EMDHS 2019 survey does not include certain variables related to contraception and lacks information on cultural factors, infrastructure, service quality, and policies in Ethiopia.

## Conclusions

The current study contributes to our understanding of the determinants of the use of modern contraceptives in married women in Ethiopia. The prevalence rate of modern contraceptives has improved slightly when compared to prior national averages; nonetheless, it remains low when compared to the national MDGS target. Age of women, region, place of resident, women education level, wealth index of the household, number of living children, and knowledge of modern contraceptive use were found as significant factors that can increase modern contraceptive use. Based on these findings, we recommend the implementation of targeted interventions tailored to underserved and vulnerable groups, particularly older women, women with lower educational attainment, and those residing in rural areas. Strengthening community awareness through focused health education campaigns can foster positive attitudes and improve knowledge about modern contraceptive use. This will aid in the development of appropriate knowledge and attitudes towards the use of modern contraception. In addition, continued efforts to improve women’s understanding of reproductive and family planning concerns should be made to improve their educational attainment. It’s also crucial to pay special attention to women between the ages of fifteen and thirty-four years.

### Recommendations

To ensure that all women are informed about modern contraceptive use, it is essential for all stakeholders, including governmental and non-governmental organizations, to educate women through various channels such as media, community initiatives, and effective counseling. In this study, we utilized logistic regression; we suggest that future research employ more robust models to examine variations, the interactions between variables, optimize parameter estimation, and assess goodness of fit.

## Data Availability

The datasets generated and/or analyzed during the current study are available in [http://dhsprogram.com/data/available-datasets.cfm](http:/dhsprogram.com/data/available-datasets.cfm).

## Abbreviations

AOR: Adjusted odds ratio
DHSS: Demographic and Health Surveillance System
DF: Degrees of Freedom
EDHS: Ethiopian demographic health survey
EMDHS: Ethiopian Mini Demographic and Health Survey
CI: Confidence interval
CSA: Central Statistical Agency
FMOH: Federal ministry of health
IUD: Intra-uterine device
LAM: lactational amenorrhea method
SPSS: Statistical Product and Service Solutions

## References

[1] Zenebe, M. & Haukanes, H. When abortion is not within reach: Ethiopian university students struggling with unintended pregnancies. *Int. J. Equity Health*. **18**, 1–13 (2019). 30691486 10.1186/s12939-019-0925-2 PMC6350354

[2] Bekele, Y. A. & Fekadu, G. A. Factors associated with unintended pregnancy in ethiopia; further analysis of the 2016 Ethiopian demographic health survey data. *BMC Pregnancy Childbirth*. **21**, 1–7 (2021). 34229647 10.1186/s12884-021-03924-0 PMC8259031

[3] Organization, W. H. Trends in maternal mortality: 1990 to 2008, (2010).

[4] Kassa, T. B., Degu, G. & Birhanu, Z. Assessment of modern contraceptive practice and associated factors among currently married women age 15–49 years in Farta district, South Gondar zone, North West Ethiopia. *J. Public. Heal*. **2**, 507–512 (2014).

[5] Hall, K. S. et al. Bad Girl and unmet family planning need among Sub-Saharan African adolescents: the role of sexual and reproductive health stigma. *Qual. Res. Med. Healthc.* **2**, 55 (2018). 30556052 10.4081/qrmh.2018.7062 PMC6292434

[6] Organization, W. H. *Family Planning Evidence Brief: Reducing Early and Unintended Pregnancies among Adolescents* (World Health Organization, 2017).

[7] Abdelbaqy, M. A. Reproductive Health in Arab Countries, Handb. Healthc. Arab World. 3–40. (2021).

[8] Hailemariam, A. The second biggest African country undergoing rapid change: ethiopia, africa’s Popul. *Search. Demogr Divid* 53–69. (2017).

[9] Medhanyie, A. A. et al. Factors associated with contraceptive use in tigray, North Ethiopia. *Reprod. Health*. **14**, 1–11 (2017). 28228141 10.1186/s12978-017-0281-x PMC5322676

[10] Hailegebreal, S. et al. Individual and community-level factors associated with modern contraceptive use among adolescent girls and young women in ethiopia: a multilevel analysis of 2016 Ethiopia demographic and health survey. *Arch. Public. Heal*. **79**, 1–12 (2021). 10.1186/s13690-021-00736-8 PMC8607592 34809708

[11] Olika, A. K., Kitila, S. B., Terfa, Y. B. & Olika, A. K. Contraceptive use among sexually active female adolescents in ethiopia: trends and determinants from National demographic and health surveys. *Reprod. Health*. **18**, 104 (2021). 34034741 10.1186/s12978-021-01161-4 PMC8146240

[12] Meselu, W., Habtamu, A., Woyraw, W. & Tsegaye, T. B. Trends and predictors of modern contraceptive use among married women: analysis of 2000–2016 Ethiopian demographic and health surveys, public heal. *Pract* **3**, 100243 (2022). 10.1016/j.puhip.2022.100243 PMC9461593 36101770

[13] Bekele, D. et al. Contraceptive prevalence rate and associated factors among reproductive age women in four emerging regions of ethiopia: a mixed method study. *Contracept. Reprod. Med.* **6**, 18 (2021). 34059143 10.1186/s40834-021-00162-9 PMC8167955

[14] Asresie, M. B., Fekadu, G. A. & Dagnew, G. W. Contraceptive use among women with no fertility intention in Ethiopia. *PLoS One*. **15**, e0234474 (2020). 32525935 10.1371/journal.pone.0234474 PMC7289351

[15] Shiferaw, S. et al. Trends in contraceptive use and distribution of births with demographic risk factors in ethiopia: a sub-national analysis. *Glob Health Action*. **8**, 29720 (2015). 26562138 10.3402/gha.v8.29720 PMC4642368

[16] Shumet, T., Geda, N. R. & Hassan, J. A. Barriers to modern contraceptive utilization in ethiopia, contracept. *Reprod. Med.* **9**, 1–12 (2024). 10.1186/s40834-024-00311-w PMC11460042 39380033

[17] Tessema, Z. T., Teshale, A. B., Tesema, G. A., Yeshaw, Y. & Worku, M. G. Pooled prevalence and determinants of modern contraceptive utilization in East africa: A Multi-country analysis of recent demographic and health surveys. *PLoS One*. **16**, e0247992 (2021). 33735305 10.1371/journal.pone.0247992 PMC7971875

[18] Götmark, F. & Andersson, M. Human fertility in relation to education, economy, religion, contraception, and family planning programs. *BMC Public. Health*. **20**, 1–17 (2020). 32087705 10.1186/s12889-020-8331-7 PMC7036237

[19] Ahinkorah, B. O. Predictors of modern contraceptive use among adolescent girls and young women in sub-Saharan africa: a mixed effects multilevel analysis of data from 29 demographic and health surveys. *Contracept. Reprod. Med.* **5**, 1–12 (2020). 33292675 10.1186/s40834-020-00138-1 PMC7678092

[20] Achana, F. S. et al. Spatial and socio-demographic determinants of contraceptive use in the upper East region of Ghana. *Reprod. Health*. **12**, 1–10 (2015). 25890034 10.1186/s12978-015-0017-8 PMC4389318

[21] Arokiasamy, P. Gender preference, contraceptive use and fertility in india: regional and development influences. *Int. J. Popul. Geogr.* **8**, 49–67 (2002).

[22] Lawson, D. W., Schaffnit, S. B., Hassan, A. & Urassa, M. Shared interests or sexual conflict? Spousal age gap, women’s wellbeing and fertility in rural Tanzania. *Evol. Hum. Behav.* **42**, 165–175 (2021).

[23] Kriel, Y. et al. Male partner influence on family planning and contraceptive use: perspectives from community members and healthcare providers in KwaZulu-Natal, South Africa. *Reprod. Health*. **16**, 1–15 (2019). 31238960 10.1186/s12978-019-0749-y PMC6593556

[24] Kabagenyi, A. et al. Barriers to male involvement in contraceptive uptake and reproductive health services: a qualitative study of men and women’s perceptions in two rural districts in Uganda. *Reprod. Health*. **11**, 1–9 (2014). 24597502 10.1186/1742-4755-11-21 PMC3946591

[25] Nkonde, H., Mukanga, B. & Daka, V. Male partner influence on women’s choices and utilisation of family planning services in Mufulira district. *Zambia Heliyon* 9 (2023). 10.1016/j.heliyon.2023.e14405 PMC10025139 36950585

[26] D’Souza, P., Bailey, J. V., Stephenson, J. & Oliver, S. Factors influencing contraception choice and use globally: a synthesis of systematic reviews. *Eur. J. Contracept. Reprod. Heal Care*. **27**, 364–372 (2022). 10.1080/13625187.2022.2096215 36047713

[27] Alspaugh, A., Barroso, J., Reibel, M. & Phillips, S. Women’s contraceptive perceptions, beliefs, and attitudes: an integrative review of qualitative research. *J. Midwifery Womens Health*. **65**, 64–84 (2020). 31135081 10.1111/jmwh.12992

[28] Silumbwe, A. et al. Community and health systems barriers and enablers to family planning and contraceptive services provision and use in Kabwe district, Zambia. *BMC Health Serv. Res.* **18**, 1–11 (2018). 29855292 10.1186/s12913-018-3136-4 PMC5984360

[29] Coleman, M. & Alonso, A. A qualitative study exploring how family planning beliefs and attitudes contribute to family planning behavior in rural, southeastern kenya: application of the social ecological model, world med. *Heal Policy*. **8**, 364–381 (2016).

[30] Berhan, Y., Ali, M., Tassew, A. & Nonogaki, A. Universal health coverage policy and progress towards the attainment of universal sexual and reproductive health and rights services in Ethiopia. *Ethiop. J. Health Sci.* **32** (2022). 10.4314/ejhs.v32i1.19 PMC8864396 35250229

[31] Kibret, M. A. & Gebremedhin, L. T. Two decades of family planning in Ethiopia and the way forward to sustain hard-fought gains! *Reprod. Health*. **19**, 124 (2022). 35698148 10.1186/s12978-022-01435-5 PMC9191530

[32] van der Heijden, L. M. J. Young Ethiopians’ Embeddedness in Migration Networks, the Role of Social Media and Their Perceptions of Western Migration Destinations, (2018).

[33] Gilano, G. et al. Understanding child wasting in ethiopia: Cross-sectional analysis of 2019 Ethiopian demographic and health survey data using generalized linear latent and mixed models. *JMIR Public. Heal Surveill*. **9**, e39744 (2023). 10.2196/39744 PMC9947770 36753309

[34] Negash, B. T., Chekol, A. T. & Wale, M. A. Modern contraceptive method utilization and determinant factors among women in ethiopia: multinomial logistic regression mini-EDHS-2019 analysis, contracept. *Reprod. Med.* **8**, 40 (2023). 10.1186/s40834-023-00235-x PMC10367398 37488640

[35] Shagaro, S. S., Gebabo, T. F. & Mulugeta, B. T. Four out of ten married women utilized modern contraceptive method in ethiopia: A multilevel analysis of the 2019 Ethiopia mini demographic and health survey. *PLoS One*. **17**, e0262431 (2022). 35030213 10.1371/journal.pone.0262431 PMC8759669

[36] Gebre, M. N. & Edossa, Z. K. Modern contraceptive utilization and associated factors among reproductive-age women in ethiopia: evidence from 2016 Ethiopia demographic and health survey. *BMC Womens Health*. **20**, 1–14 (2020). 32216823 10.1186/s12905-020-00923-9 PMC7098091

[37] Senaviratna, N. & Cooray, T. Diagnosing multicollinearity of logistic regression model. *Asian J. Probab. Stat.* **5**, 1–9 (2019).

[38] Geremew, A. B. & Gelagay, A. A. Modern contraceptive use and associated factors among married women in Finote Selam town Northwest ethiopia: a community based cross-sectional study. *Women’s Midlife Heal*. **4**, 1–8 (2018). 10.1186/s40695-018-0044-z PMC6297988 30766723

[39] Kettema, W. G., Aynalem, G. L., Yismaw, A. E. & Degu, A. W. Modern contraceptive utilization and determinant factors among street Reproductive-Aged women in Amhara regional state zonal towns, North West ethiopia, 2019: Community‐Based study. *Int. J. Reprod. Med.* **2020**, 7345820 (2020). 33354561 10.1155/2020/7345820 PMC7737461

[40] Csace, I. Ethiopia demographic and health survey 2016, Addis Ababa, Ethiop. Rockville, Maryland, USA CSA ICF. 1–551. (2016).

[41] Debebe, S., Limenih, M. A. & Biadgo, B. Modern contraceptive methods utilization and associated factors among reproductive aged women in rural Dembia district, Northwest ethiopia: community based cross-sectional study. *Int. J. Reprod. Biomed.* **15**, 367 (2017). 29202123 PMC5605858

[42] Hamdalla, T., Arega, A. & Markos, T. Prevalence and associated factors of modern contraceptive utilization among married women in reproductive age group in misha woreda Hadiya zone, South Ethiopia. *J. Women’s Heal Care* 6 (2017).

[43] Beson, P., Appiah, R. & Adomah-Afari, A. Modern contraceptive use among reproductive-aged women in ghana: prevalence, predictors, and policy implications. *BMC Womens Health*. **18**, 1–8 (2018). 30253759 10.1186/s12905-018-0649-2 PMC6156857

[44] Kaggwa, E. B., Diop, N. & Storey, J. D. The role of individual and community normative factors: a multilevel analysis of contraceptive use among women in union in Mali. *Int. Fam Plan. Perspect.* 79–88. (2008). 10.1363/ifpp.34.079.08 18644759

[45] Bogale, B., Wondafrash, M., Tilahun, T. & Girma, E. Married women’s decision making power on modern contraceptive use in urban and rural Southern Ethiopia. *BMC Public. Health*. **11**, 1–7 (2011). 21595897 10.1186/1471-2458-11-342 PMC3114727

[46] Islam, M. R. & Thorvaldsen, G. Family planning knowledge and current use of contraception among the Mru Indigenous women in bangladesh: a multivariate analysis. *Open. Access. J. Contracept.* 9–16. (2012).

[47] Oyedokun, A. O. Determinants of contraceptive usage: lessons from women in Osun state, Nigeria. *J. Hum. Soc. Sci.* **1**, 1–14 (2007).

[48] Kayembe, P. K., Fatuma, A. B., Mapatano, M. A. & Mambu, T. Prevalence and determinants of the use of modern contraceptive methods in kinshasa, Democratic Republic of congo. *Contraception* **74**, 400–406 (2006). 17046382 10.1016/j.contraception.2006.06.006

[49] Prata, N. et al. Varying family planning strategies across age categories: differences in factors associated with current modern contraceptive use among youth and adult women in luanda, Angola. *Open. Access. J. Contracept.* 1–9. (2016). 10.2147/OAJC.S93794 PMC5683147 29386932

[50] Ahinkorah, B. O. Predictors of unmet need for contraception among adolescent girls and young women in selected high fertility countries in sub-Saharan africa: a multilevel mixed effects analysis. *PLoS One*. **15**, e0236352 (2020). 32760153 10.1371/journal.pone.0236352 PMC7410238

[51] Lakew, Y., Reda, A. A., Tamene, H., Benedict, S. & Deribe, K. Geographical variation and factors influencing modern contraceptive use among married women in ethiopia: evidence from a National population based survey. *Reprod. Health*. **10**, 1–10 (2013). 24067083 10.1186/1742-4755-10-52 PMC3850415

[52] Shiferaw, S. et al. Does proximity of women to facilities with better choice of contraceptives affect their contraceptive utilization in rural ethiopia?? *PLoS One*. **12**, e0187311 (2017). 29131860 10.1371/journal.pone.0187311 PMC5683563

[53] Wang, W. *How Family Planning Supply and the Service Environment Affect Contraceptive Use: Findings from Four East African Countries* (International Health and Development, ICF International, 2012).

[54] Cavallaro, F. L., Benova, L., Macleod, D., Faye, A. & Lynch, C. A. Examining trends in family planning among harder-to-reach women in Senegal 1992–2014. *Sci. Rep.* **7**, 41006 (2017). 28106100 10.1038/srep41006 PMC5247687

[55] SOLANKE, B. L. Socio-demographic factors associated with unmet need for family planning among women who experienced pregnancy termination in Nigeria. *Afr. J. Psychol. Stud. Soc. Issues*. **19**, 112–125 (2016).

[56] Endriyas, M. et al. Contraceptive utilization and associated factors among women of reproductive age group in Southern nations nationalities and peoples’ region, ethiopia: cross-sectional survey, mixed-methods. *Contracept. Reprod. Med.* **2**, 1–9 (2017). 10.1186/s40834-016-0036-z PMC5683467 29201415

[57] Kebede, A., Abaya, S. G., Merdassa, E. & Bekuma, T. T. Factors affecting demand for modern contraceptives among currently married reproductive age women in rural Kebeles of Nunu Kumba district, oromia, ethiopia, contracept. *Reprod. Med.* **4**, 1–15 (2019). 10.1186/s40834-019-0103-3 PMC6894206 31844553

[58] de Vargas Nunes, C., Coll, F., Ewerling, F., Hellwig, A. J. D. & De Barros Contraception in adolescence: the influence of parity and marital status on contraceptive use in 73 low-and middle-income countries. *Reprod. Health*. **16**, 1–12 (2019). 30791914 10.1186/s12978-019-0686-9 PMC6383262

[59] Geda, N. R. & Lako, T. K. A population based study on unintended pregnancy among married women in a district in Southern Ethiopia. *J. Geogr. Reg. Plan.* **4**, 417 (2011).

[60] Kibret, A., Bayu, H. & Merga, M. Prevalence of unintended pregnancy and associated factors among pregnant women attending antenatal clinics in Debre-markos town, North West Ethiopia 2012. *J. Women’s Heal Care*. **4**, 420–2167 (2015).

[61] Gite, A., Liulseged, N. & Seyife, H. Unintended pregnancy: magnitude and associated factors among pregnant women in Arba minch town, Gamo Gofa zone, ethiopia, 2015. *Reprod. Syst. Sex. Disord* ; **5** (193), (2016). (n.d.).

[62] Mosha, P. E., Mgimwa, C. A. & Msuya, S. M. Assessment of knowledge and perception towards modern contraceptives use among women of reproductive age in Mtwivila. *Tanzan. Sci. J. Public. Heal*. **5**, 335–340 (2017).

